# Spatial cognition and science achievement: The contribution of intrinsic and extrinsic spatial skills from 7 to 11 years

**DOI:** 10.1111/bjep.12211

**Published:** 2018-01-22

**Authors:** Alex Hodgkiss, Katie A. Gilligan, Andrew K. Tolmie, Michael S. C. Thomas, Emily K. Farran

**Affiliations:** ^1^ UCL Institute of Education University of London UK; ^2^ Birkbeck College London UK

**Keywords:** spatial cognition, science, development, science, technology, engineering, and mathematics

## Abstract

**Background:**

Prior longitudinal and correlational research with adults and adolescents indicates that spatial ability is a predictor of science learning and achievement. However, there is little research to date with primary‐school aged children that addresses this relationship. Understanding this association has the potential to inform curriculum design and support the development of early interventions.

**Aims:**

This study examined the relationship between primary‐school children's spatial skills and their science achievement.

**Method:**

Children aged 7–11 years (*N *=* *123) completed a battery of five spatial tasks, based on a model of spatial ability in which skills fall along two dimensions: intrinsic–extrinsic; static–dynamic. Participants also completed a curriculum‐based science assessment.

**Results:**

Controlling for verbal ability and age, mental folding (intrinsic–dynamic spatial ability), and spatial scaling (extrinsic–static spatial ability) each emerged as unique predictors of overall science scores, with mental folding a stronger predictor than spatial scaling. These spatial skills combined accounted for 8% of the variance in science scores. When considered by scientific discipline, mental folding uniquely predicted both physics and biology scores, and spatial scaling accounted for additional variance in biology and variance in chemistry scores. The children's embedded figures task (intrinsic–static spatial ability) only accounted for variance in chemistry scores. The patterns of association were consistent across the age range.

**Conclusion:**

Spatial skills, particularly mental folding, spatial scaling, and disembedding, are predictive of 7‐ to 11‐year‐olds’ science achievement. These skills make a similar contribution to performance for each age group.

## Background

Large‐scale longitudinal studies spanning the past 50 years provide convincing evidence that spatial ability in adolescence predicts later science, technology, engineering, and mathematics (STEM) achievement (Lubinski & Benbow, 2006; Wai, Lubinski, & Benbow, 2009). In addition to often cited examples of scientific discoveries resulting from creative spatial thought, a growing body of research with adults and adolescents highlights a more specific link between spatial ability and various aspects of science learning (e.g., Kozhevnikov & Thornton, 2006). However, in contrast to the spatial ability and mathematics literature (e.g., Mix *et al*., 2016), the relationship between spatial ability and science learning in younger children has been largely neglected.

A deeper understanding of this relationship at an earlier stage of development is important because it has implications for early curriculum design, informs the development of spatial training interventions, and has the potential to support learners when they are at more advanced stages of science education. The focus of this study was therefore on the relationship between different aspects of spatial ability and scientific achievement in primary‐school children. Below, we present a summary of current knowledge of spatial ability and science learning before discussing the relationship between these two domains.

### Overview of spatial ability

Spatial ability, which relates to ‘the location of objects, their shapes, their relation to each other, and the paths they take as they move’ (Newcombe, 2010, p. 30), has long been recognized as an ability partly independent of general intelligence, reasoning, and verbal ability (Hegarty, 2014; Rimfeld *et al*., 2017). As well as being distinct from other cognitive abilities, spatial thought itself has often been conceptualized in a multidimensional fashion: as consisting of several separate but correlated skills.

Two broad categories of multidimensional models have emerged: ones based in the psychometric tradition (Carroll, 1993; Lohman, 1988) and other more theoretically driven models (e.g., Uttal *et al*., 2013). This study adopts a theoretical model, proposed by Uttal and colleagues (Newcombe & Shipley, 2015; Uttal *et al*., 2013), based on top‐down understanding of spatial skills, drawing upon developments in cognitive neuroscience. The model primarily distinguishes between intrinsic and extrinsic spatial abilities, mapping onto a within‐object and between‐object classification, respectively. Intrinsic/extrinsic skills are further categorized as either static or dynamic abilities; dynamic abilities include transformation or movement.

*Intrinsic–static* skills involve the processing of objects or shapes, or parts of objects or shapes, without further transformation. Tasks that measure this skill often require this processing to occur amidst distracting background information. For example, in disembedding tasks, participants search for a specified 2D shape in a larger distracting image. *Intrinsic–dynamic* skills, in contrast, involve the processing and manipulation or transformation of objects or shapes. Mental folding and mental rotation fit into this category. *Extrinsic–static* skills require the processing and encoding of the spatial relations between objects, without further transformation of these relations. The extrinsic–static category includes spatial alignment or reasoning about spatial correspondence, an example of which is the ability to find corresponding locations between shapes of equal proportion but differing sizes (scaling and map use). *Extrinsic–dynamic* skills involve the transformation of the relationship between objects, or the relationship between objects and frames of reference. Spatial perspective taking, in which a participant visualizes a change in their relationship to an object and is asked what an object or objects would look like from a different viewpoint, is an extrinsic–dynamic skill.

The model is supported by research indicating that object‐based spatial ability (intrinsic) is partially dissociated from environmental (extrinsic) spatial ability (Hegarty, Montello, Richardson, Ishikawa, & Lovelace, 2006). The intrinsic–extrinsic dimension is also supported by the finding that mental rotation (intrinsic–dynamic) and perspective taking (extrinsic–dynamic) are associated with different patterns of brain activation (Zacks, Vettel, & Michelon, 2003) and are also psychometrically distinct (Hegarty & Waller, 2004).

### Science learning

The goal of science is to extend our knowledge of the world. ‘Science’ therefore refers to both the existing body of knowledge that we have about the world and the activities and processes by which this knowledge comes about (Zimmerman, 2000). Engaging in science in part involves understanding and applying factual knowledge and conceptual understanding of the theories that exist about the phenomena around us. Scientific knowledge is commonly organized by discipline, for example, physics, and specific subtopics within these domains, such as the subtopic of electricity. In addition to this, science involves specific reasoning, strategies, and investigation skills which are directed towards discovery and changes to the theories we have about the world (Zimmerman, 2000). The ability to form and evaluate scientific hypotheses is one example of an important scientific reasoning skill.

In this study, a curriculum‐based approach to science assessment was adopted. The UK science curriculum includes the previously outlined aspects of factual knowledge, conceptual understanding, and scientific investigation (Department for Education, 2013). It also emphasizes that ’working scientifically … must always be taught through and clearly related to substantive science content in the programme of study’ (Department for Education, 2013, p. 5.). Science achievement was therefore assessed using a composite assessment of factual knowledge, conceptual understanding, and investigation skills taught in the age range of interest. A curriculum‐based approach has the advantage that it covers the breadth of knowledge and skills children learn in the classroom. Such an approach has also been successfully adopted in the past, for example, in studies investigating the role of executive functions on children's performance in standardized science assessments (Jarvis & Gathercole, 2003; St Clair‐Thompson & Gathercole, 2006).

### Spatial skills and science

Spatial skills may particularly support learning, problem‐solving, and reasoning within conceptual science areas that have a clear spatial–relational basis (e.g., astronomy and mechanics). Table 1 provides other hypothetical examples of how the different spatial skills as outlined by Uttal *et al*. (2013) might be applied to different scientific activities (Rule, 2016).

**Table 1:** Examples of Uttal *et al*.'s (2013) spatial skill categories in relation to scientific activities, Rule (2016)

Uttal *et al*. (2013) category	Description of category	Scientific field	Examples of scientific activities
Intrinsic–static	Processing of objects/shapes without transformation	Geology	Identifying rocks and rock formations by colour, texture, grain size, and visual patterns
Intrinsic–dynamic	Processing and manipulation or transformation of objects/shapes	Chemistry	Checking the symmetry of atoms in a crystal structure by imagining them moving across mirror planes or rotating around an axis
Extrinsic–static	Encoding of the spatial relations between objects without transformation	Chemistry	Comparing the crystal structures of a compound with and without a substituted element
Extrinsic–dynamic	Transformation or updating of the relationship between objects	Astronomy	Locating a near‐earth asteroid's path through time and its distances from the earth as both move along different paths

Most prior research with adults points to spatial visualization skills as being related to science learning. Spatial visualization involves mentally transforming object‐based spatial information and is assessed through intrinsic–dynamic spatial skills such as mental rotation. Existing research with adults suggests a link between intrinsic–dynamic spatial skills and conceptual understanding in aspects of biology (Garg, Norman, Spero, & Maheshwari, 1999), chemistry (Stull, Hegarty, Dixon, & Stieff, 2012), and physics (Kozhevnikov & Thornton, 2006). For example, in Stull *et al*. (2012) spatial ability, as measured through 3D object visualization, correlated with undergraduate students’ ability to translate between different diagrammatic representations of chemical structures. There is also some evidence linking adults’ chemistry performance to disembedding (intrinsic–static) spatial skills (Bodner & McMillen, 1986) and undergraduate's geoscience understanding to multiple‐object (extrinsic–dynamic) spatial skills (Sanchez & Wiley, 2014). However, no research to‐date has addressed other skills, such as extrinsic–static scaling ability, in relation to science learning.

### Spatial skills and science in children

Research relating spatial ability and science learning in younger children is sparse, and some studies that have addressed this have done so only in relation to visual–spatial working memory (VSWM) or a limited range of spatial skills. Two studies (Jarvis & Gathercole, 2003; St Clair‐Thompson & Gathercole, 2006) focused on 11‐year‐olds’ achievement in UK national standardized science tests in relation to working memory. The findings of both studies pointed towards the VSWM task as being predictive of performance in science. However, because these tasks are designed to test both the visual and spatial aspects of spatial cognition, complex working memory span tasks often confound object/visual, and location/spatial skills. It is therefore not possible to determine the extent to which the associations reported relate to the more intrinsic and extrinsic, or static and dynamic, aspects of the spatial task.

A few studies to date have examined children's science performance and learning in relation to other spatial skills (e.g., Harris, 2014; Mayer, Sodian, Koerber, & Schwippert, 2014; Tracy, 1990). Tracy (1990), for example, found that 10‐ to 11‐year‐olds in a higher spatial ability grouping outperformed those in a lower spatial ability grouping on a standardized science measure. However, this study did not include any other non‐spatial cognitive measures and therefore did not discount such cognitive factors as an alternative explanation. It also used a composite spatial measure. One more recent study that did compare different spatial ability measures found that mental folding accuracy, but not mental rotation accuracy, predicted 5‐year‐old's understanding of force and motion, but this finding was limited to intrinsic–dynamic skills (Harris, 2014).

### Changes in the relationship between spatial ability and science at different stages of learning

Spatial skills may be more important for individuals at an earlier stage of learning than those in later stages (Uttal & Cohen, 2012). During initial learning, or for individuals with lower levels of domain‐specific knowledge, a learner may use spatial processing to establish mental maps and models, or to problem solve (Mix *et al*., 2016). In line with this, for example, Hambrick *et al*. (2012) found that spatial ability interacted with adults’ level of geological knowledge in a geology task in which participants inferred the geologic structure of a mountain range. Specifically, spatial ability was more predictive of performance for participants who had lower levels of geologic knowledge, whereas for those with more domain‐specific knowledge, spatial skills were less important.

Developmentally, this hypothesis is also supported by the finding that mental folding ability, an intrinsic–dynamic skill, predicts children's, but not adult's, understanding of forces (Harris, 2014). One possible interpretation of this finding is that younger children must actively visualize the effects of forces to make predictions, whereas adults rely more on knowledge of forces and their effects, which has accumulated over time. The above findings suggest that spatial skills may therefore play a more important role in science achievement for younger compared with older children; however, this has yet to be addressed empirically.

### Current study

The aim of this study was to examine the relationship between various dimensions of 7‐ to 11‐year‐old's spatial skill and their performance in a science assessment, which covered aspects of biology, chemistry, and physics knowledge as well as scientific investigation skills within these areas. School year groups in the UK are further grouped into larger curriculum‐linked ‘key stages’. Children in years 3 to year 6 (aged 7–11) are grouped into ‘Key Stage 2’. We therefore sampled children from each year group within Key Stage 2, which meant that the children in the sample were working towards the same overall curriculum objectives. Using a range of ages, we also aimed to determine whether this relationship was moderated by age. Given the dearth of literature on the relationship between children's spatial skills and science reasoning, it is difficult to make specific predictions. Based on the findings of Harris (2014), we predicted that, minimally, intrinsic–dynamic skills would be related to science performance, and this relationship may be stronger for younger children.

## Methods

### Participants

Participants were recruited from a large London primary school. The most recent percentage of children eligible for free school meals in the school, which provides an indicator of levels of socioeconomic disadvantage, was 19%, compared to a national average of 14% (Department for Education, 2017). The ethnicity of the school population was 44% Asian, 29% White, 13% Black, and 14% mixed/other. Ethical approval was granted by the University College London, Institute of Education, Research Ethics Committee. Three pupils did not go on to complete the study because they were unsuitable due to having a special educational need or an insufficient level of English. Due to missing data caused by technical failure, five participants did not have a full set of scores available for analysis. Four of these participants were missing data from one task only, and to maximize statistical power, their missing scores (two British Picture Vocabulary Scale‐III [BPVS‐III] scores, one mental folding score and one perspective taking score) were estimated by calculating the mean for their respective year group and replacing their missing score with the mean value. The fifth participant was missing several variables and was excluded from the analysis. Thus, four participants were excluded in total. The final sample therefore consisted of 123 participants in years 3–6. A summary of the age and gender of participants by year group is provided in Table 2.

**Table 2:** Summary of descriptive statistics and demographics for each year group

Year group	Number of participants in group	Mean age (years)	*SD* age	Gender (% female)
Year 3	32	8.03	0.28	44
Year 4	31	8.97	0.33	53
Year 5	31	9.95	0.33	47
Year 6	29	11.01	0.30	43

### Measures

#### Spatial measures overview

The choice of measures of spatial ability was based on the model by Uttal *et al*. (2013). One measure was included for each of the categories outlined except for the intrinsic/dynamic category, where two spatial measures (mental folding and mental rotation) were included. We chose to include both measures because there are key differences between them, despite falling into the same category in Uttal *et al*.'s (2013) model (Newcombe, 2016). Mental rotation is a rigid, intrinsic/dynamic transformation, and folding is a non‐rigid, intrinsic/dynamic transformation (Atit, Shipley, & Tikoff, 2013). In rigid transformations, such as mental rotation, the distances between every pair of points of an object are preserved (Atit *et al*., 2013). During a non‐rigid transformation, such as mental folding or bending, the distances between points of a shape change as the transformation occurs. Additionally, prior research by Harris (2014) found mental folding, and not mental rotation, to be a predictor of force understanding.

#### Intrinsic–static spatial measure: Children's Embedded Figures Task

The Children's Embedded Figures Task (Karp, Konstadt, & Witkin, 1971) consists of complex figures in which a simple form is embedded. The test was administered in accordance with the manual. Children were shown an image constructed of geometric shapes and asked to locate either a simple house or tent shape ‘hidden’ within the image. Children were shown this shape in a cardboard form, which matched the shape hidden in the image. For the first part of the test (11 items), children located a triangular tent shape within each image and for the other half of the test (14 items) children located a house shape. For the first three items in both the tent and the house trials, the child retained the cardboard shape to assist their search. The experimenter removed the shape thereafter. Accuracy was recorded on a laptop. When the child believed they had successfully located the hidden figure, they pressed a designated button on the laptop. The child outlined the location of the hidden shape to indicate their response. The researcher then pressed one of two buttons to record accuracy, depending on whether the child was correct or incorrect.

#### Intrinsic–dynamic spatial measure: monkey mental rotation

**Figure 1:**
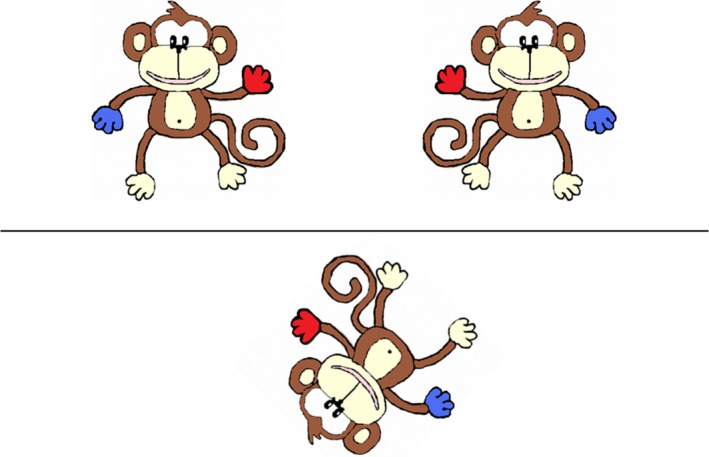
Example 135° trial from the mental rotation task. Children selected which monkey at the top matched the monkey at the bottom.

In this task (Broadbent, Farran, & Tolmie, 2014), children were shown two upright cartoon monkeys, above a horizontal line, on a computer screen, and one monkey below a line which was rotated by varying degrees (0°, 45°, 90°, 135°, 180°) (Figure 1). One monkey above the horizontal line had a blue left hand and a red right hand, and the other monkey had the reverse pattern and was a mirror image of the other. Children were asked which of the two upright monkeys at the top of the screen matched the rotated monkey at the bottom of the screen. Children gave their response by pressing one of two preselected keys on a computer. This task began with four practice items, in which the monkey below was not rotated (0° degree trials); answers to these practice items were checked by the researcher. Only participants who correctly answered 50% or more of the practice items on their first attempt correctly proceeded to the main trials. All participants answered 50% or more correctly on their first attempt. Participants then progressed to 40 experimental trials (8 × 0° trials, 8 × 45° trials, 8 × 90° trials, 8 × 135° trials, and 8 × 180° trials). Accuracy and response times were recorded by the computer via the child's keyboard responses to each item.

#### Intrinsic–dynamic spatial measure: Mental Folding Test for Children

**Figure 2:**
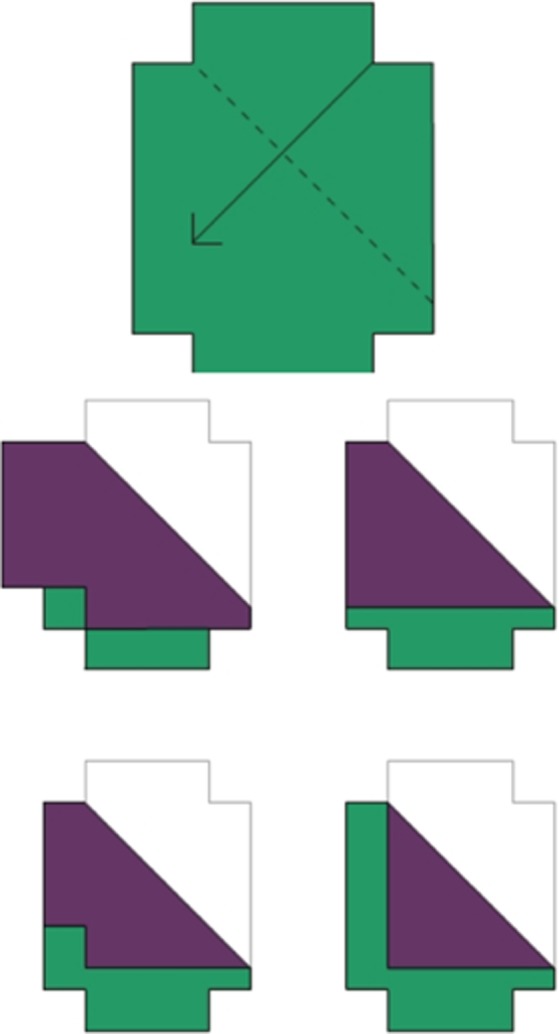
Mental folding trial. Children were asked to imagine folding the shape at the top, as directed by an arrow and a dashed fold line, and to then select one shape at the bottom which showed the shape after the fold.

This mental folding task (Harris, Newcombe, & Hirsh‐Pasek, 2013) required children to imagine folds made to a piece of paper, without physical representation of the folding action itself. Children were shown a shape at the top of a computer screen (Figure 2) which contained a dotted line and an arrow. The dotted line represented the imaginary fold line, and the arrow indicated where the paper should be folded to. Beneath this item on the screen, children were shown four images of how the item at the top might look after being folded at the dotted line, only one of which was correct. Children first completed two practice items (one of which they could use a physical card version to check their answer). Answers to practice questions were checked by the researcher, and if a child had an incorrect answer, they were given one further attempt of each practice item. The majority of participants passed the practice trials on their first attempt, and all passed on the second, if needed. The experimental trials then began, where children had 14 items to work through. The test progressed automatically as the child clicked one of the four images at the bottom of the screen. Accuracy was recorded on the computer through the child's mouse response to each item.

#### Extrinsic–static spatial measure: spatial scaling

**Figure 3:**
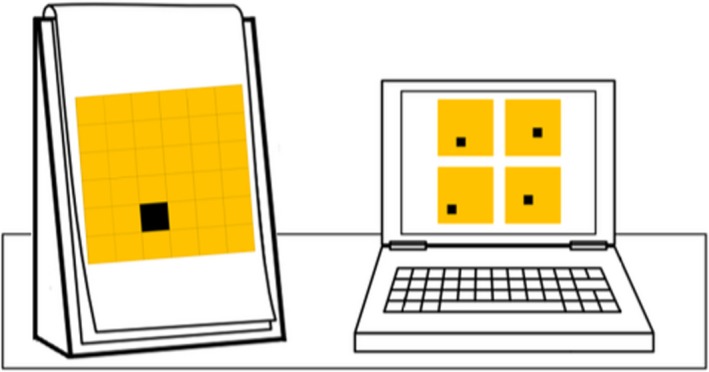
Spatial scaling layout and example trial (6 × 6 grid and 1:2 scaling). Children were asked to determine which map on the computer had the target location in the same position as the printed map, to the left of the computer.

Our novel spatial scaling task (Gilligan, Hodgkiss, Thomas, Tolmie, & Farran, manuscript submitted) was developed from similar tasks by Frick and Newcombe (2012) and Möhring, Newcombe, and Frick (2016). Children were required to find equivalent corresponding locations on two maps, when one was varied in size relative to the other by a predetermined scale factor. Participants were presented with four treasure maps on a touch screen computer, each of which had one black square (the treasure location) at different locations for each map (Figure 3). Next to the computer, children were presented with one printed treasure map, mounted in an A3 ring bound pad. The child's task was to determine which of the four maps on the computer screen had the black treasure location positioned in the same place as the larger printed map. Only one of the computer maps contained the treasure location in the same position as the printed map. The other three, incorrect, options were created uniformly for each trial.

The larger printed maps were either unscaled (1:1; 7 cm × 7 cm), or scaled to either 1:2 (14 cm × 14 cm) or 1:4 (28 cm × 28 cm), relative to the maps on the computer (7 cm × 7 cm each). Nine (of 18) items contained grids which separated the map into 6 × 6 (larger) grid sections, requiring gross level acuity, whereas the other nine items contained grids which separated the map into 10 × 10 (smaller) sections, requiring fine level acuity. Although both the computer and the printed maps were separated into grid sections, the grid lines were visible only on the larger printed maps. Six items were presented at each scale factor. Participants first completed two practice items, which needed to be answered correctly before proceeding, after which, they completed the main 18 trials of the test. If participants did not get the answer correct, they were given feedback and one further chance to complete the practice item. Only participants who correctly answered 50% or more of the practice items on their first attempt correctly continued to the main trials. All participants answered 50% or more correctly on their first attempt.

#### Extrinsic–dynamic spatial measure: photo spatial perspective taking task

**Figure 4:**
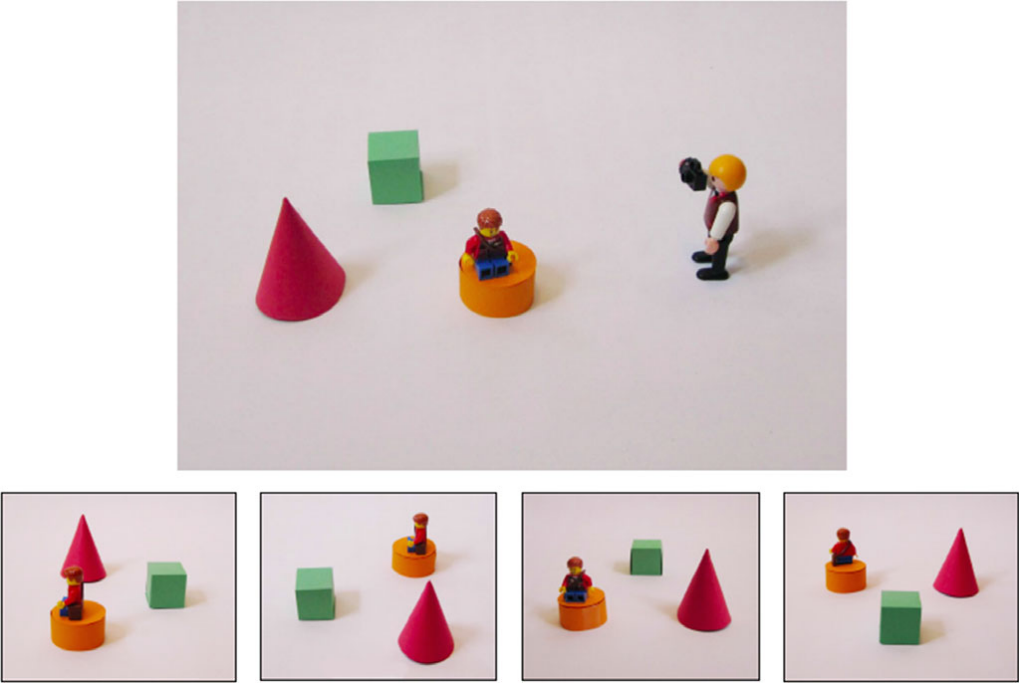
Spatial perspective taking trial (three objects and 90° angular difference to child's perspective). Children selected which photograph at the bottom showed what the photograph would look like taken by the character above.

This task (Frick, Möhring, & Newcombe, 2014) involved spatial perspective taking in which children were required to visualize what photographs would look like when taken from cameras placed at different positions and angles relative to their viewpoint. The child first completed four practice questions involving physical Playmobil characters. The experimenter placed two characters, who were each holding a camera, next to two objects, in a specified arrangement on a table. The child was then shown four photographs of the objects, taken from the perspective of one of the characters, and asked which of the two characters could have taken the photograph. The characters were rearranged, and the question was asked again with new photographs. Participants then completed one additional practice question on a laptop computer, which showed a Playmobil character taking a photograph of two objects from the same perspective as the child (0° angular difference trial). The child was shown four possible photographs that could have been taken by the character. The child then selected the correct option of four by pressing a touch screen computer (Figure 4). If a child made an error on the practice items, they were given a maximum of one additional attempt at each practice item. Feedback was given on each practice item. Few children made errors on the first attempt and all passed on their second, if one was needed.

On passing the practice questions, the task then continued with the main trials. These varied per the number of objects in the layout (1, 2 or 3) and the angular difference between the photographer's and the child's perspective (0°, 90° or 180°). The task consisted of two blocks of nine trials; each of the three angular differences was presented once for one, two, and three object trials. The first block progressed with all one object trials first, followed by two object trials and finally all three object trials. The second block was reversed such that it began with three objects, working back to one object only. Accuracy was recorded on the computer through the child's touch screen response to each item.

#### Science assessment

The science assessment consisted of two paper‐based tests, which children completed in two sessions, in class groups, under the supervision of the researcher. All questions were read to participants by the researcher. The assessment was a composite, curriculum‐based measure, and questions were taken from a selection of past science UK standardized (‘SATS’) test papers designed to assess science achievement in this age range (e.g., Qualifications and Curriculum Authority (QCA), 2009). The test included approximately equal numbers of biology, chemistry, and physics focused questions on topics appropriate to this curriculum stage (‘Key Stage 2’: age 7–11).

Each paper had a total possible score of 50 marks leading to a total science mark of 100. The assessment included questions which varied in difficulty. The difficulty level of each question was determined by the categorization given in the testing materials, which is linked to curriculum target descriptors. Paper one contained questions of low to medium demand and paper two contained questions of high demand. Paper one contained 11 questions and paper two contained 10 questions. Each question focused on one subtopic, for example, magnets (see Table 3 for topics). Each question was divided into several subitems (approximately 4 per question); see Appendix. Some items were more factual/recall based (e.g., what is the function of the roots of a plant?), others required more conceptual understanding (explain why the bigger sail makes the boat go faster) or were more problem‐solving‐based. Some items in the context of hypothetical experiments, related to the subtopic, required investigation skills (e.g., identify a prediction). There was a mixture of free response and multiple choice items throughout. The two papers had good levels of internal consistency as measured by Cronbach's α = .841 (paper 1) and α = .794 (paper 2), across all items. A second coder scored a random 10% of the first and second papers and demonstrated a high degree of inter‐rater reliability with the first coder (*r *=* *.99, *p* = <.001).

**Table 3:** Summary of subtopics included in the science assessment

Biology	Chemistry	Physics
Plants (functions of parts, seed dispersal, life cycle)	Properties of materials	Light (shadows, reflections)
Human skeleton	Changing state (condensation, melting, and evaporation)	Sun, earth, and moon
Human growth and development	Reversible and non‐reversible changes	Gravity and forces
Classifying and sorting animals	Rocks	Electricity

#### Control variables

Vocabulary is highly correlated with overall general intellectual ability (Sattler, 1992); therefore, the BPVS‐III (Dunn, Dunn, Styles, & Sewell, 2009) was included as a measure of verbal ability, but also serves as an estimate of general intelligence. The experimenter read a word to the child, who then matched it to one of four pictures. The words became increasingly difficult and testing was discontinued when the child made eight errors within one set.

### Procedure

Children first completed two paper‐based science assessments, in two sessions. Sessions lasted approximately 45 min each. Science assessments were administered by the researcher in class groups, within the child's own classroom. Spatial ability was then assessed within two separate sessions. Children were first tested in a computer‐based group of no more than eight children, lasting approximately 35 min, where they completed the mental folding task and the monkey mental rotation task. Group testing sessions were supervised by at least two researchers. The BPVS, Children's Embedded Figures Test, spatial perspective taking task, and scaling task were then completed in an individual testing session with the researcher, which lasted approximately 45 min per child. The order of tasks in the individual sessions and group testing session was counterbalanced. Within each of the group and individual testing sessions, children also completed additional mathematics tasks, not reported here (see Gilligan, Hodgkiss, Thomas & Farran, manuscript in preparation).

## Results

### Descriptive statistics

A total science score was calculated by totalling the participants’ scores across both paper 1 and paper 2. A total for biology, chemistry, and physics questions across both papers was also calculated. Mean accuracy on the individual spatial ability tasks, mean reaction time, and accuracy for the mental rotation task and mean science scores are reported in Table 4.

**Table 4:** Descriptive statistics for science total scores, British Picture Vocabulary Scale raw scores, and spatial measures

Measure	*M*	*SD*	Range
Correct overall science score (100)	43.97	14.60	7–75
Correct overall science score, Y3 (100)	35.75	10.87	7–51
Correct overall science score, Y4 (100)	41.42	14.78	14–72
Correct overall science score, Y5 (100)	47.26	14.31	18–71
Correct overall science score, Y6 (100)	52.24	13.31	21–75
Correct overall biology score (36)	18.63	6.17	3–33
Correct overall chemistry score (32)	13.11	5.03	1–26
Correct overall physics score (32)	12.91	5.56	2–29
I‐D (mental rotation accuracy) (40)	33.06	5.8	6–40
I‐D (mental rotation reaction time)	4059.77	1186.1	892.16–6644.95
I‐D (mental folding accuracy) (14)	9.36	2.71	0–14
I‐S (children's embedded figures accuracy) (25)	13.64	4.26	5–23
E‐S (scaling task accuracy) (18)	11.59	3.23	4–18
E‐D (spatial perspective taking accuracy) (18)	12.22	3.77	5–18

Notes

I‐D = intrinsic–dynamic; I‐S = intrinsic–static; E‐S = extrinsic–static; E‐D = extrinsic–dynamic; Y3 = year 3; Y4 = year 4; Y5 = year 5; Y6 = year 6.

Maximum possible score in parentheses.

Reaction times for correct responses only were considered for mental rotation. This type of rotation task is a variation of a chronometric mental rotation task where children are shown pairs of objects and asked whether they are the same or mirror images. Accuracy and response time is typically considered as a marker of individual differences for these types of mental rotation task (Jansen, Schmelter, Quaiser‐Pohl, Neuburger, & Heil, 2013). Response times 2.5 *SD*s above or below the mean of each cell (angle of rotation) were excluded from the analysis (Whelan, 2008). Values for each participant were calculated by finding the overall mean reaction time for each degree of rotation (45°, 90°, 135°, 180°).

### Correlation analysis

Bivariate correlations were also analysed between the predictive variables (BPVS, age, and spatial ability measures) and the dependent variables (total science score and biology, chemistry and physics subscores), which are reported in Table 5. Partial correlations, controlling for age and BPVS raw scores, between each of the spatial measures and each of the science totals, are reported in the lower triangle of Table 5.

**Table 5:** Bivariate and partial correlations between study variables

	1	2	3	4	5	6	7	8	9	10	11	12
1. British Picture Vocabulary Scale (BPVS) raw score	–	436**	.747**	.636**	.656**	.630**	.272**	.109	.291**	.197*	.401**	.420**
2. Age	–	–	.453**	.333**	.421**	.530**	.196*	−.053	.197*	.257**	.259**	.367**
3. Science overall total	–	–	–	.881**	.866**	.880**	.289**	.006	.466**	.366**	.507**	.504**
4. Biology total	–	–	–	–	.756**	.710**	.264**	.074	.418**	.307**	.460**	.480**
5. Chemistry total	–	–	–	–	–	.714**	.238**	−.032	.351**	.319**	.429**	.423**
6. Physics total	–	–	–	–	–	–	.278**	−.043	.395**	.351**	.425**	.469**
7. Mental rotation (acc)	–	–	.117	.117	.066	.119	–	.274**	.294**	.068	.221*	.417**
8. Mental rotation (RT)			−.092	.015	−.119	−.112	–	–	−.041	−.100	−.027	−.005
9. Mental folding	–	–	.384**	.311**	.211*	.276*	–	–	–	.407**	.408**	.456**
10. Embedded figures	–	–	.308**	.230*	.227*	.250**	–	–	–	–	.308**	.360**
11. Scaling	–	–	.329**	.285**	.225*	.222*	–	–	–	–	–	.518**
12. Perspective taking	–	–	.280*	.295**	.178	.229*	–	–	–	–	–	–

Notes

Acc = accuracy; RT = reaction time. Upper triangle shows zero‐order correlations, and lower triangle shows partial correlations between spatial measures and the science total score, controlling for BPVS raw score and age in months.

*p < 0.05; **p < 0.01.

Controlling for these covariates, neither mental rotation accuracy nor response time correlated with any science variables. The mental folding task, the embedded figures task, and the scaling task had small to moderately sized partial correlations (range: .211 < *r* < .384) with total science scores and biology, chemistry, and physics scores. Perspective taking scores also had small to moderately sized positive partial correlations (range: .229 < *r* < .295) with all science variables other than chemistry scores, where there was no significant correlation.

### Regression analysis

Regression analyses were run for overall science scores and for biology, chemistry, and physics scores. There were no significant gender differences in any science scores (*p* > .05 for all); therefore, participants were treated as one group in the subsequent regression analyses. A hierarchical and stepwise approach was taken to determine the amount of variance in science outcomes that was accounted for by participants’ spatial ability, taking into account the covariates (age and BPVS raw score). In all regression models, covariates were added hierarchically first. Betas reported refer to the final models (Tables 6, 7, 8, 9).

**Table 6:** Multiple regression analysis predicting science total score

Predictor	*b*	β	*p*	∆*F*	Sig ∆*F*	*R* ^2^	*R* ^2^∆
Step (1) Age (months)	.130	.122	.044	31.27	<.001	.21	.21
Step (2) British Picture Vocabulary Scale raw score	.412	.567	<.001	106.16	<.001	.58	.37
Step (3) Folding (I‐D)	1.135	.211	.001	20.62	<.001	.64	.06
Step (4) Scaling (E‐S)	.735	.162	.010	6.79	.010	.66	.02

Notes

Betas refer to values when all predictors are entered into the final model. The Sig ∆*F* is the *p* value of the change in *F* for each step of the regression model.

**Table 7:** Multiple regression analysis predicting biology score

Predictor	*b*	β	*p*	∆*F*	Sig ∆*F*	*R* ^2^	*R* ^2^∆
Step (1) Age (months)	.015	.034	.648	15.10	<.001	.11	.11
Step (2) British Picture Vocabulary Scale raw score	.152	.495	<.001	60.38	<.001	.41	.30
Step (3) Folding (I‐D)	.448	.197	.008	12.77	.001	.47	.06
Step (4) Scaling (E‐S)	.331	.173	.025	5.13	.025	.49	.02

Note

Betas refer to values when all predictors are entered into the final model. The Sig ∆*F* is the *p* value of the change in *F* for each step of the regression model.

**Table 8:** Multiple regression analysis predicting chemistry score

Predictor	*b*	β	*p*	∆*F*	Sig ∆*F*	*R* ^2^	*R* ^2^∆
Step (1) Age (months)	.045	.122	.103	26.09	<.001	.18	.18
Step (2) British Picture Vocabulary Scale raw score	.129	.517	<.001	60.52	<.001	.45	.28
Step (3) Embedded Figures (I‐S)	.167	.141	.046	6.47	.012	.48	.03
Step (4) Scaling (E‐S)	.229	.147	.049	3.95	.049	.50	.02

Notes

Betas refer to values when all predictors are entered into the final model. The Sig ∆*F* is the *p* value of the change in *F* for each step of the regression model.

**Table 9:** Multiple regression analysis predicting physics score

Predictor	*b*	β	*p*	∆*F*	Sig ∆*F*	*R* ^2^	*R* ^2^∆
Step (1) Age (months)	.121	.297	<.001	47.28	<.001	.28	.28
Step (2) British Picture Vocabulary Scale raw score	.121	.439	<.001	44.98	<.001	.48	.20
Step (3) Folding (I‐D)	.428	.209	.002	9.78	.002	.52	.04

Notes

Betas refer to values when all predictors are entered into the final model. The Sig ∆*F* is the *p* value of the change in *F* for each step of the regression model.

Entered in the first step of each model, age in months significantly predicted overall scores and scores for individual science areas. Age remained a significant predictor in the final model for overall science scores and physics scores. However, age was not significant in the final model for biology or chemistry. Participants’ BPVS raw score was entered in the second step of each model and was a significant predictor of all science outcomes. BPVS scores remained a significant predictor in all of the final models.

Following entry of age and BPVS scores, we then considered the predictive role of the spatial ability measures. All spatial predictors found to be significantly associated with the respective science score in the prior partial correlation analysis were entered together as a block using forward stepwise entry. Forward stepwise entry was used due to the inter‐relatedness of the spatial variables, and because we had no strong theoretical predictions about the basis for a hierarchical ordering of variables within this block.

The forward entry of spatial measures predicting overall science score retained mental folding and spatial scaling. Mental folding accounted for an additional 6% of the variance in total science score, ∆*F*(1,119) = 20.62, *p* = <.001, and the scaling task then accounted for a further 2% of the variance in total science scores, ∆*F*(1,118) = 6.79, *p* = .010, above the covariates. In the final model, which accounted for 65% of the variance in total science scores (adjusted *r* ^2^), mental folding was a stronger predictor (β = .211) than scaling (β = .162).

Forward entry of the spatial measures predicting biology scores also retained mental folding and spatial scaling. After step 2, mental folding accounted for an additional 6% of the variance in biology scores, ∆*F*(1,119) = 12.77, *p* = .001, and the spatial scaling task accounted for an additional 2% of the variance in biology scores ∆*F*(1,118) = 5.13, *p* = .025. The overall model accounted for 47% of the variance in biology science scores (adjusted *r* ^2^). Mental folding was a stronger predictor (β = .197) than scaling (β = .173) in the final model.

The embedded figures task was retained as a significant spatial predictor of chemistry scores accounting for a further 3% of the variance in chemistry scores, ∆*F*(1,119) = 6.47, *p* = .012, above the covariates. In addition, the scaling task was also retained as a predictor of chemistry scores, which accounted for an additional 2% of the variance, ∆*F*(1,118) = 3.95, *p* = .049. The final model accounted for 48% of the variance in participants’ chemistry total score (adjusted *r* ^2^). The two spatial skills in this model had similarly sized β coefficients: embedded figures, β = .141; scaling β = .147. Mental folding was the only retained predictor of the physics scores. It was entered in step 3, and it accounted for an additional 4% of the variance in physics scores, ∆*F*(1,119) = 9.78, *p* = .002. The final model accounted for 51% of the variance in physics scores (adjusted r^2^).

To determine whether age interacted with any of the spatial ability measures, and therefore whether this pattern varied across the age groups, a further four models were constructed in which the covariates were again entered in step 1, followed by the spatial ability measures found to be significant for that science score, followed by an interaction term (age in months × spatial measure). No significant age interactions were found (*p* > .05 for all).

## Discussion

The aim of the current study was to examine the contribution of spatial skills to primary‐school children's performance in a curriculum‐based science assessment. The study revealed overall that spatial ability is a predictor of 7‐ to 11‐year‐olds’ science achievement. After controlling for receptive vocabulary, which provided an estimate of general intelligence, spatial ability accounted for an additional 8% of the variance in total science scores. This builds upon longitudinal research linking spatial ability to STEM outcomes in adults (Lubinski & Benbow, 2006; Wai *et al*., 2009) as well as correlational research associating spatial ability to various aspects of science learning in adults (e.g., physics problem‐solving: Kozhevnikov & Thornton, 2006). It also builds on research linking VSWM to general science performance in 11‐year‐olds (Jarvis & Gathercole, 2003; St Clair‐Thompson & Gathercole, 2006) and spatial skills to 5‐year‐olds’ force and motion understanding (Harris, 2014) in two main ways. First, it investigated a broader range of spatial skills and science topic areas. Second, it sampled a wider age range of children within one study to investigate possible developmental changes.

It is first interesting to note that both an intrinsic and an extrinsic spatial skill uniquely predicted overall science scores. This suggests that both within‐object and between‐object spatial skills support children's science reasoning and supports the broad dissociation between intrinsic and extrinsic spatial skills (Hegarty *et al*., 2006). Considering the role of specific spatial skills, the results revealed that mental folding, an intrinsic–dynamic spatial skill, was the strongest spatial predictor of total science scores. This general finding builds on past research linking mental folding ability to adult science outcomes (e.g., Baker & Talley, 1972).

Mental folding also emerged as the strongest spatial predictor of biology scores. This is the first study to date linking mental folding ability to biology with children. The ability to flexibly visualize, maintain, and manipulate spatial information may be related to mental model construction and utilization (Lohman, 1996). A mental model (Johnson‐Laird, 1983; Zwaan & Radvansky, 1998) is a structural analog that contains spatial and conceptual relations of a process or situation. Children may construct spatially grounded mental models of problem‐solving questions, which include relational aspects of the problem, and then manipulate these mental models to solve them. This has been proposed in mathematics research with children (e.g., Rasmussen & Bisanz, 2005). Additionally, the representations children have for domain‐specific concepts within biology may be spatially grounded. For example, many of the plant‐related questions involve knowledge and understanding of plant anatomy and function, which may be related to one another in mental model format. When recalling the function of roots, children may recall a spatial mental model of a plant, which includes spatial–relational information about the location and structure of different parts of the plant.

Mental folding also predicted physics scores, a finding which builds on the work of Harris (2014), who found that mental folding predicted 5‐year‐olds’ force and motion understanding. Recall that the mental folding task requires non‐rigid, dynamic visualization. The spatial skills required to accurately visualize paper folds may support children in, for example, visualizing and predicting the dynamic effects of forces acting on objects, or the general dynamic transfer of energy, which is central to physics topics. More specifically, spatial visualization skills may enable children to mentally simulate actions and processes, such as reasoning about the way two magnets react to each other.

After controlling for BPVS scores, mental rotation was not a predictor of science achievement, despite it falling into the same Uttal *et al*. (2013) category as mental folding; this was also found by Harris (2014) in relation to children's force and motion understanding in 5‐year‐olds. There are several plausible reasons for this. First, as previously described, rotation is a rigid transformation and folding is a non‐rigid transformation. In contrast to rotation, where the relationship between all points of the object is preserved, folding creates two separate areas, and the spatial relations between these areas must be maintained as the shape is folded. It is plausible that the additional spatial requirements of the folding task supported more complex visualization between multiple elements in the science assessment. In addition, there are also possible limitations with the rotation task itself. The task uses the same monkey stimuli throughout, with the choice stimuli having the same pattern of blue and red hands, rather than using a range of animals, as is the case with other 2D rotation tasks (e.g., Neuburger, Jansen, Heil, & Quaiser‐Pohl, 2011). It is possible that this resulted in children of this age range using a rule‐based strategy (i.e., if the monkey's right hand is red in one stimuli, then it will appear to be on the left side on the rotated version), rather than an analog, rotation‐based strategy. Finally, research to date with adults and adolescents linking mental rotation to science achievement uses abstract 3D cube mental rotation, in contrast to the 2D animal stimuli used in the current study. Although children up to the age of 10 have difficulty with 3D rotation in its traditional format (Jansen *et al*., 2013), a 3D mental rotation task with tangible objects has more recently been developed which is suitable from 4 years (Hawes, LeFevre, Xu, & Bruce, 2015). Future work could further investigate the possible influence of stimuli type and test format.

Spatial scaling, an extrinsic/static skill, also emerged as a predictor of total scores, biology scores, and chemistry scores. To our knowledge, this is the first study to link extrinsic–static spatial skills with science achievement. The National Research Council's report ‘A Framework for K‐12 Science Education’ (National Research Council, 2012) also identifies scaling within the core theme ‘scale, proportion, and quantity’. It emphasizes that understanding relative magnitude and scale is essential for science; for instance, children must learn to appreciate how systems and processes vary significantly in size (e.g., a cell vs. an organism). Taking a chemistry topic example from the current study, when understanding states of matter, children link how a liquid behaves at the observable *macroscopic* scale with the molecular processes at the *microscopic* scale. The report also identifies that children need to confidently move back and forth between representational models of different scales (e.g., for biology: a diagrammatic representation and a life‐sized human skeleton model). Switching between scaled models is a central component of the scaling task used in the current study.

The embedded figures task, an intrinsic–static spatial skill, was a significant predictor of chemistry scores only. This builds on prior work which found a relationship between this task and adults’ chemistry performance (Bodner & McMillen, 1986). Intrinsic–static spatial skills relate to form perception and the processing of objects without further transformation. Several of the chemistry items include diagrams which require processing subparts of objects (e.g., three beakers, each with four ice cubes, which either have 1, 2, or 3 layers of insulation). The visual discrimination between the diagrams may support problem‐solving needed for this type of question.

Interestingly, biology emerged as the discipline area which was most strongly predicted by spatial ability generally, despite the fact that it is not generally thought of as a spatially demanding area, relative to physics, for example. Although there are examples of spatial ability being related to biology learning in adults (e.g., learning anatomy: Lufler, Zumwalt, Romney, & Hoagland, 2012), in the Wai *et al*. (2009) longitudinal study, spatial ability in adolescence was predictive of outcomes in physics, engineering and chemistry, but not biology. Although biological concepts may not immediately appear as spatial as other areas, the abstract spatial representations used to organize and classify (e.g., classification keys: binomial, branching tree diagrams used to identify species) may be spatially demanding. It is possible that there is a greater utilization of these kinds of spatial representations for children than for adults.

Models predicting overall science score and performance in each area of science were consistent across development. It had been predicted that spatial skills may contribute more to science performance for younger children, suggesting that as domain‐specific knowledge increases, spatial abilities play less of a role in science (e.g., Hambrick *et al*., 2012); however, this was not upheld in the data. Such a hypothesis is based on the idea that older or more experienced learners can apply knowledge more readily without having to process spatially. For example, this prediction would suggest that spatial visualization would not be a strong predictor of questions where children determined the direction of a force acting on an object because they would simply ‘know’ the answer, without having to visualize it. However, this was not the case. The assessment covered a wide range of topics and it may be that, although the older children were indeed more experienced in science, their in‐depth knowledge (i.e., knowledge they could recall at the time of doing the assessment) may have been restricted to the topic or topics they have recently covered in class, for example. Furthermore, the children were all in the same academic Key Stage; with a wider age range, above 12 years possibly, developmental changes may have been observed.

There are also limitations with the study. First, although we included the BPVS as a measure of verbal ability, we did not include a measure of non‐verbal reasoning ability. It is possible that the relationships observed may be partly accounted for by aspects of the task that involve fluid intelligence or non‐verbal reasoning, in addition to the spatial skill measured. Second, the nature of the composite science assessment used includes aspects of factual knowledge, conceptual understanding, and problem‐solving. Dividing outcome measures into these subskills is a possibility for future research.

Relatedly, items also differed in the extent to which they required participants to use overtly spatial representations, such as diagrams. The observed relationship between spatial skills and science achievement may be driven by items which included spatial representations such as these. This is supported by a prior study demonstrating the effectiveness of a science curriculum which included spatial skills training in the form of diagram reading instruction (Cromley *et al*., 2016). The training was most effective for science post‐test items in which interpretation of the diagram was particularly important in answering the question because the diagrams had been used to relate novel curriculum content. That is, the students had not been exposed to the topic or diagram previously in class and the question answer could therefore be derived from interpretation of the diagram alone. Many diagrams in the current study also had a degree of novelty because they were often included to accompany previously unseen problems and scenarios. Future research could compare the contribution of spatial skills to performance on items which rely on diagrams to varying degrees.

The results observed in the current study have implications for interventions to support children's science learning. Given evidence that spatial skills are malleable (Uttal *et al*., 2013), the finding that spatial scaling, mental folding, and disembedding predict children's science achievement suggests that they are good candidates for spatial training. Long‐term interventions involving the training of multiple spatial skills, embedded within the curriculum, may be a particularly effective approach (see Hawes, Moss, Caswell, Naqvi, and MacKinnon (2017) for a mathematics example). Furthermore, interventions to support children's spatial thinking skills could lead to additional long‐term benefits for science achievement and engagement.

## Appendix

Example biology items from paper 1. Item (a) requires conceptual understanding whereas (b) is more knowledge/recall based.
